# Hospitals and Medical Teaching

**Published:** 1920-02-21

**Authors:** 


### The Hospital

The Workers' Newspaper of Administrative Medicine and Institutional Life, Administration, National Insurance and Health.

No. 1759, Vol. LXVII. SATURDAY, FEBRUARY 21, 1920. PRICE TWOPENCE

### HOSPITALS AND MEDICAL TEACHING.

Since the close of active military operations the medical profession individually and collectively have passed through some remarkable changes of outlook, but meanwhile one particular development has occurred which should have more far-reaching effects than possibly even the most optimistic have as yet realised. This is the new attitude of the general public towards the problem of medical education. The layman is rapidly coming to appreciate it as a matter not of interest to the medical schools alone, but as one of vital importance to his family and himself. Last week we commented upon the valuable suggestions offered by Mr. E. W. Morris, the House Governor of the London Hospital. The revived attention which has been once more given to the idea of city hospitals working on the principle of casualty clearing stations, with correspondingly wider developments of their whole organisation, is all in tune with the prevailing spirit of progress.

This turn of opinion has already been of undoubted assistance by at least accelerating many of the more recent teaching reforms in hospitals having a preponderance of laymen on their Boards of Governors, and typical of the times is the new agreement between St. Mary's Hospital and the Paddington Board of Guardians. In future the clinical material to be found in the Paddington Infirmary becomes available for instructional purposes to the medical students of St. Mary's, and although the idea itself cannot be described as new, the practice of it certainly is, and it is indeed a matter for satisfaction that the possibilities of undergraduate instruction in infirmaries have at last received official and practical demonstration.

Another point of obvious importance is that by such correlation the public itself gains greatly. The even approximate conversion of a Poor-Law Infirmary into a modern hospital with modern equipment and greater resources both in staff and material provides not only increased opportunity to perfect the training of their future medical advisers, but also places a definite increase of first-class hospital accommodation at the masses' disposal.

But let us turn back to the case of other medical establishments, other non-educational hospitals and infirmaries. A very great proportion of them have no direct or practical affiliation to teaching colleges, and there is a liability for both the local authorities, and the country as a whole, to lose sight of their educational functions and possibilities.

In America there has recently developed a strong movement towards hospital standardisation inaugurated by the American College of Surgeons. It is suggested that institutions should be investigated to see whether they come up to the standards required of them by the public at large as well as by the medical profession. It is stated that these standards require that the staff of the hospital shall recognise their duty and their obligation as teachers in order that the institution may fulfil that all-important function of extending medical knowledge with resultant benefit to the community. Their point is that every hospital, whether connected with a medical school or not, has an educative duty to perform.

Here as in America it is an obvious advantage to the hospital itself if it be so fortunate as to have a flourishing collegiate connection, and, as already explained, the public are beginning to appreciate this. The present financial difficulties of the hospital world in general are a matter of obvious and intimate concern to the general laity, and, therefore, schemes for extension and affiliation might expect a sympathetic reception. Even without such organisation, however, there is an individual obligation for every hospital properly to fulfil some educational function, be it directed to the doctors, the nursing profession, or the communities around them. How many are doing this as it should be done, or making fullest use of such opportunities as exist? An increasing but at present still small proportion.

All hospitals may be "teaching hospitals" in one sense or another, and governing bodies must realise the importance of the educational aspect. We think that the one-time apparent supremacy of Germany in the world of medicine, the financial strength of her hospitals and clinics, was largely due to their wide advertisement as teaching centres and the deliberate fashion in which a supposed educational element was fostered. This is, however, one aspect of the subject; in this country we are already making amends for previous slackness in provision of graduate education on the large scale. But on the other hand, let us remember that even the local institution can set the standard of medical practice for the professional element in its vicinity, and, what is more important, it can direct and encourage much of that public enlightenment in the essentials of healthy and hygienic living, which is at the moment of such vital importance to the community. Clinical research, too, can be accomplished with success only in the actual hospital or in close connection with the advantages it provides. Every hospital is in truth a mine of treasure in scientific facts which still await discovery, and the need for taking the utmost practical advantage of possibilities in even the smallest institution is a definite requirement of these times. Perhaps we shall in future see a gradual achievement of these ideals coming as a direct result of the impending solution to the financial difficulties of the voluntary hospital system, the object for which we are now, as in the past, so gladly and earnestly striving.

